# Effect of Composite Core Materials on Fracture Resistance of Endodontically Treated Teeth: A Systematic Review and Meta-Analysis of In Vitro Studies

**DOI:** 10.3390/polym13142251

**Published:** 2021-07-09

**Authors:** Maciej Zarow, Marzena Dominiak, Katarzyna Szczeklik, Louis Hardan, Rim Bourgi, Carlos Enrique Cuevas-Suárez, Juan Eliezer Zamarripa-Calderón, Naji Kharouf, Dimitar Filtchev

**Affiliations:** 1 Private Practice and Postgraduate Course Center NZOZ SPS Dentist, pl. Inwalidow 7/5, 30-033 Kraków, Poland; 2 Department of Dental Surgery, Silesian Piast Medical University, ul. Krakowska 26, 50-425 Wrocław, Poland; Marzena.dominiak@umed.wroc.pl; 3 Department of Integrated Dentistry, Jagiellonian University Medical College-Montelupich 4, 31-155 Cracow, Poland; k.szczeklik@uj.edu.pl; 4 Department of Restorative Dentistry, School of Dentistry, Saint-Joseph University, Beirut 1107 2180, Lebanon; louis.hardan@usj.edu.lb (L.H.); rim.bourgi@net.usj.edu.lb (R.B.); 5 Dental Materials Laboratory, Academic Area of Dentistry, Autonomous University of Hidalgo State, Circuito Ex Hacienda La Concepción S/N, San Agustín Tlaxiaca 42160, Hgo., Mexico; cecuevas@uaeh.edu.mx (C.E.C.-S.); eliezerz@uaeh.edu.mx (J.E.Z.-C.); 6 Department of Biomaterials and Bioengineering, INSERM UMR_S 1121, Strasbourg University, 67000 Strasbourg, France; 7 Department of Prosthetic Dental Medicine, Faculty of Dental Medicine, Medical University of Sofia, 1000 Sofia, Bulgaria; d.filchev@fdm.mu-sofia.bg

**Keywords:** composite core material, composite resin, dentistry, endodontically treated tooth, fracture resistance, restoration

## Abstract

Various material properties are involved in the success of endodontically treated restorations. At present, restorative composites are commonly employed as core build-up materials. This study aimed to systematically review the literature to assess the effect of using composite core materials on the in vitro fracture of endodontically treated teeth. Two different reviewers screened the literature, up to June 2021, in five distinct electronic databases: PubMed (MedLine), Scopus, Scielo, ISI Web of Science, and EMBASE. Only in vitro studies reporting the effect of the use of composite core materials on the fracture resistance of endodontically treated teeth were included. A meta-analysis was carried out using a software program (Review Manager v5.4.1; The Cochrane Collaboration, Copenhagen, Denmark). The risk of bias in each study was assessed following the parameters of another systematic review. A total of 5016 relevant papers were retrieved from all databases. After assessing the title and abstract, five publications remained for qualitative analysis. From these, only three studies remained for meta-analysis. The fracture strength of endodontically treated teeth where a core build-up composite was used was statistically significantly higher than the control (*p* = 0.04). Most of the analyses showed a high heterogenicity. The in vitro evidence suggests that the composite core build-up with higher filler content tended to improve the fracture resistance of the endodontically treated teeth, in comparison with conventional composite resins. This research received no external funding. Considering that this systematic review was only carried out on in vitro papers, registration was not performed. Furthermore, there were no identified clinical studies assessing core build-up materials; therefore, more well-designed research on these materials is needed.

## 1. Introduction

After root canal treatment, dental practitioners are faced with the task of restoring the tooth. Restoring the endodontically treated tooth is a subject that has been evaluated and discussed widely in the dental literature [1,2]. The endodontically treated tooth, after access cavity preparation, shaping procedure, and obturation steps, represents a challenge to dental practitioners, due to the loss of the tooth structure, altered physical characteristics, dehydration, and impaired neurosensory feedback mechanism [3]. Despite this, integral rehabilitation, including esthetic, functional, and structural aspects, is critical to ensuring a successful restorative outcome [4].

A common method to restore the endodontically treated teeth is the use of a post and core, onto which a full crown is cemented [5,6,7]. The post is a restorative material placed in the canal root, and its primary function is to aid in the retention of restoration and protect the tooth by dissipating or distributing forces along the tooth [8]. Endodontic posts can be pre-formed or custom made; metallic and non-metallic; esthetic and non-esthetic [9]. Among the different type of endodontic posts, the use of fiber-reinforced composite posts has increased, due to their favorable physical properties, such as a high tensile strength and good fatigue resistance. These types of post can minimize the possibility of root fracture and display significantly higher survival rates [10,11,12].

In combination with a fiber-reinforced composite post, a composite core build-up material is often used to restore the coronal portion of the teeth, in order to achieve a retention and resistance form for the preparation [13]. Restorative composites are usually used as core build-up materials, making it possible to perform the preparation after curing [14]. Despite this, it is important to note that there are many commercially available resin composites, which are specifically designed for core build-up. These materials are formulated with increased content and more types of filler, to provide them with higher strength and easier manipulation [15,16].

Despite the straightforward application of core build-up composite resins, scientific evidence that could guide the decision of clinicians when considering the use of these materials instead of conventional composite resins is scarce. Hence, the present study aimed to assess the effect of using composite core materials on the in vitro fracture of endodontically treated teeth by systematically reviewing the literature. The null hypothesis was that the fracture of endodontically treated teeth restored with core build-up composite resins is similar to that of endodontically treated teeth restored with conventional composite resins.

## 2. Materials and Methods

The present systematic review and meta-analysis was reported following the PRISMA 2020 guidelines [17], using the following PICOS framework: population, endodontically treated teeth; intervention, application of core composite for core build-up; control, application of conventional or bulk-fill composite for core build-up; outcomes, fracture resistance; study design, in vitro studies. The research question was: “Does the application of core composite materials improve the fracture resistance of human endodontically treated teeth?”

### 2.1. Literature Search

The literature search was systematically accomplished by two independent reviewers (L.H. and R.B.) up to 7 June 2021 (considering unlimited publication years). Five distinct electronic databases were screened: PubMed (MedLine), Scopus, Scielo, Embase, and ISI Web of Science, in order to identify the articles that could be included. The keywords and search strategy implemented in PubMed were depicted in Table 1. The search strategy used in Scopus, Scielo, EMBASE, and ISI Web of Science databases is presented in the Supplementary Material (Tables S1–S4). A hand-search of the reference lists of included manuscripts was also performed to identify supplementary studies. After the initial screening, all papers were imported into Mendeley Desktop 1.17.11 software (Glyph & Cog, LLC, London, UK) to eliminate duplicates.

### 2.2. Study Selection

Two reviewers (R.B. and L.H.) independently assessed the titles and abstracts of all manuscripts. Papers were selected for full-text review according to the following inclusion criteria: (1) in vitro studies evaluating the effect of the use of composite core materials on the fracture resistance of endodontically treated teeth; (2) included a control group in which bulk-fill or conventional composite resin was applied following the manufacturers’ instructions; (3) included a group where core build-up composite resin was used; (4) evaluated the fracture resistance of endodontically treated teeth restored with composite resins and core build-up composite resins; (5) included mean and standard deviation data in N. Only studies written in the English language were considered for this review. Papers that involved endodontically treated bovine teeth were excluded. Clinical trials, case reports, pilot studies, case series, and reviews were also excluded. Full copies of all of the potentially relevant manuscripts were analysed. Papers that had insufficient data in the title and abstract to make a clear decision regarding their inclusion were selected for a full reading. Any disagreement regarding the eligibility of the included manuscripts was decided through consensus and discussion with a third author (C.E.C.-S.). Only papers that satisfied all the listed eligibility criteria were included in the review.

### 2.3. Data Extraction

The data of interest from the included papers were extracted by means of a standardized sheet (Microsoft Office Excel software, Microsoft Corporation, Redmond, WA, USA). These data contained the author and year of publication, composite resins used, core-build up composite used, outcomes evaluated, and main results. When papers that revealed this information in graph format, the data of interest were retrieved by calculation using WebPlotDigitizer 4.0 software (Austin, TX, USA).

### 2.4. Quality Assessment

The risk of bias parameters for each included study were evaluated by two authors (M.Z. and D.F.), according to another systematic review [18]. The risk of bias in was assessed according to the description given for the following parameters: random sequence generation; single-operator protocol implementation; the presence of a control group; blinding of the testing machine operator; standardization of the sample preparation; failure mode evaluation; use of the materials following manufacturer’s instructions; clarification of the sample size calculation. If the examined parameter was reported by the author, the study received a “YES”. On the other hand, if information was missing, the parameter received a “NO.” Risk of bias from each study was classified according to the sum of the “YES” answers received: 1 to 3 corresponded to a high, 4 to 6 medium, and 7 to 8 to a low risk of bias.

### 2.5. Statistical Analysis

A meta-analysis was performed using the Review Manager v5.4.1 software program (The Cochrane Collaboration, Copenhagen, Denmark). Only studies classified as having a low or medium risk of bias were included in a meta-analysis. The analysis was performed using the random-effects model, and pooled effect estimates were obtained by comparing the standardized mean difference in fracture resistance between endodontically treated teeth restored with core-build up composites and endodontically treated teeth restored with conventional composites. A level of significance lower than 0.05 was considered statistically significant. Heterogeneity was tested using the Cochran Q test and the inconsistency I2 test.

## 3. Results

**Figure 1:**
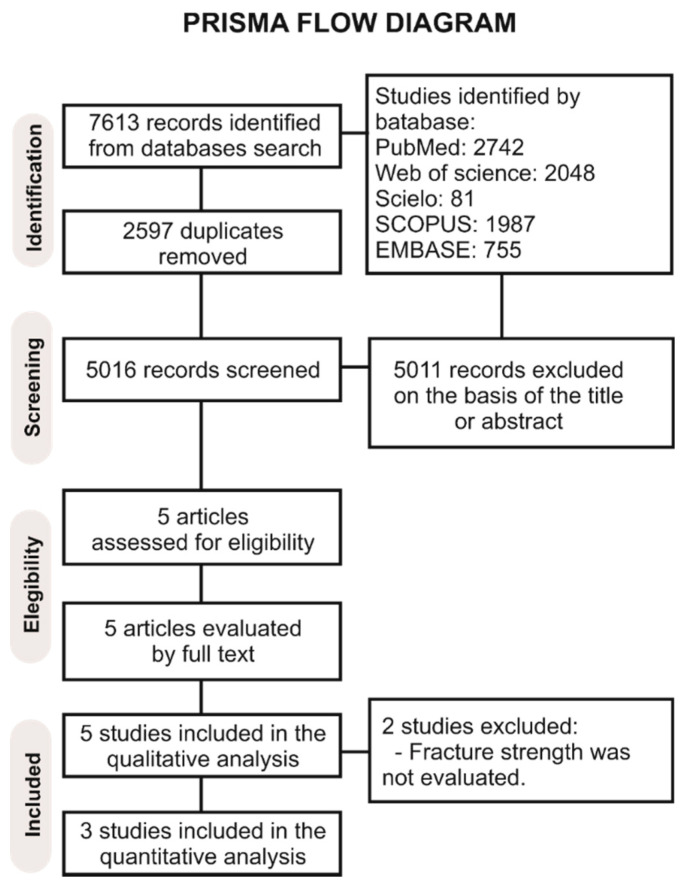
Prisma flow diagram of the study.

A total of 7613 papers was recognized in all databases. A flowchart describing the study selection process according to the PRISMA Statement is shown in Figure 1. After removing the duplicates, the literature search retrieved 2597 manuscripts for the initial examination. Then, 5016 studies were excluded after reviewing the titles and abstracts, leaving a total of five studies to be assessed by full-text reading [19,20,21,22,23]. Of these, two studies were not considered in the qualitative analysis [21,22], leaving a total of three articles [19,20,23] that were used in the meta-analysis; the reasons for exclusion are given in the PRISMA flow diagram.

**Figure 2:**
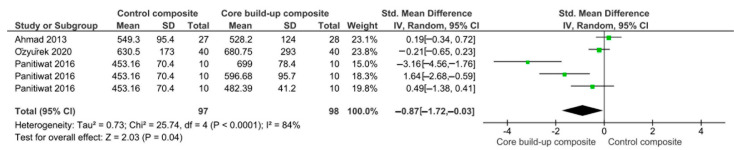
Results of the meta-analysis of the fracture strength of endodontically treated teeth, restored with a core-build up composite or a conventional composite. Fracture strength was higher when the endodontically treated teeth were restored with a core-build up composite (*p* = 0.04).

The meta-analysis suggested that the fracture strength of endodontically treated teeth was statistically significantly higher than the control when a core build-up composite was used (*p* = 0.04) (Figure 2).

A qualitative synthesis of the manuscripts considered in this systematic review is summarized in Table 2.

Considering the parameters for the risk-of-bias assessment of the included studies, all the studies were classified as having medium risk of bias; therefore, they were considered suitable for meta-analysis (Table 3). However, the majority of studies failed to report the following items: sample size calculation, single operator, and operator-blinded.

## 4. Discussion

A systematic review and meta-analysis were conducted to analyze the effect of composite core materials on the fracture resistance of endodontically treated teeth, in comparison with conventional composite resins. The overall analysis revealed that the fracture resistance of endodontically treated teeth improved when the core build-up composite was used. Therefore, the null hypothesis tested in this study was rejected, as there were significant differences in fracture resistance when using diverse composite build-up materials.

It should be noted that the strength of the composite core build-up is a main factor in the achievement of a long-lasting restoration when the remaining tooth structure is limited [24]. As stress was engaged on the core material, a higher strength material was needed to resist fracture load [23]. Furthermore, the fracture resistance between composite resins might be linked to the material properties in terms of the bonding’s ability to post and dentin, strength, mode of polymerization, and rigidity [25].

Resin composites mostly constitute a combination of an organic matrix, a bisphenol A-glycidyl methacrylate (Bis-GMA) compound, and filler particles. However, other composites with higher filler content are used for core build-up [23,24]. In this study, the higher fracture strength obtained using core-build up materials could be related to their filler content [19]. An increase in the filler content resulted in an increase in the flexural modulus. Furthermore, an increase in the core materials’ modulus resulted in an increase in fracture resistance [26]. This could explain the findings of this study, as a previous paper showed that the stiffness of a core material within an elastic range could be indicated by the flexural modulus which, in turn, reflected the longevity and strength of the restoration. In this respect, the ideal distribution of the masticatory forces to the root and post could be achieved using a core build-up material with the same dentin substrate modulus [27].

Aside from the effect of fillers on the fracture resistance of a pulpless tooth, the bonding ability of composite materials plays an essential role in the strength-promotion highlighted in this meta-analysis. Since the bonding agent was applied before core build-up, according to the manufacturer’s instructions, incompatibility between materials was avoided [28]. Another potential explanation for the higher strength obtained is the fact that the core material, when used with low consistency, achieved a better integration with the post, due to the fact that air bubbles and voids were minimized within the core-post interface or the core [29]. In this manner, clinicians should consider that the performance of core materials relies on their formulation. A more successful, endodontically treated restoration could be obtained by selecting a suitable composite material to use with the post. Accordingly, the findings in this manuscript suggest that dentist use core composite build-up materials in case the tooth has coronal loss. The methodological quality assessment revealed that all the included manuscripts were classified as having a medium risk of bias, which denotes that the quality of the evidence for the assessed outcome might be high. In relation to this, it should be emphasized that the sample size calculation and the operator blinding parameters were not stated in most of the investigated papers, and failure to define these factors could expand the likelihood of the performance and detection of bias.

Some unexplored aspects could have influenced the results of the present report. The presence of nanofillers in the polymeric composite resin or the restoration type could have an influence on mechanical properties and fracture strength; therefore, future studies could also take these variables into account [30,31]. Furthermore, resin composites specifically designed for core build-up are formulated with an increase in fillers for higher strength and easy manipulation, which could also affect the results [32].

The findings of this review must be considered with caution since, in clinical situations, a wet environment, and masticatory stresses lead to a rapid core-post debonding. Teeth may tolerate these forces with the aid of periodontal tissues. Furthermore, high heterogeneity was found in all the comparisons, which warranted the careful interpretation of these results. Future research must be conducted, particularly randomized controlled clinical trials, with the purpose of providing better insights into the performance of core build-up composites in the clinical success of an endodontically treated tooth. Moreover, the evidence should be directed towards testing other core build-up materials, with different properties.

From this review, in vitro evidence was analyzed regarding the composite core build-up materials used in the literature to obtain a high fracture resistance to pulpless tooth. It should be emphasized that the main reason for the failure of endodontically treated teeth is related to materials such as crown debonding, post-debonding, or root fracture. It is important to mention that the core material is a critical component of overall success in the restoration of endodontically treated teeth, especially when used with post [12,33]. Consequently, it seems that establishing a higher fracture resistance to pulpless tooth is crucial in the long-term clinical success of restorative treatment.

As randomized clinical trials assessing this variable are scant, the best evidence available to date comes from in vitro studies, such as those collected by this systematic review. Future randomized clinical trials studying the clinical performance of endodontically treated teeth, restored using resin composites specifically designed for core build-up, are highly desired.

## 5. Conclusions

In conclusion, within the limitation of the long distance between laboratory studies and clinical randomized evaluations, the in vitro evidence implies that composite core build-up with higher filler content tends to improve the fracture resistance of endodontically treated teeth in comparison with conventional composite resins.

## Figures and Tables

**Table 1:** Keywords and search strategy used in PubMed.

Search Strategy	
**#1**	Pulpless tooth OR pulpless teeth OR root filled tooth OR root filled teeth OR endodontically treated tooth OR endodontically treated teeth OR devital tooth OR devital teeth OR tooth nonvital OR root canal treatment OR root filling OR endodontical treated teeth OR endodontics OR root canal therapy OR tooth root OR nonvital OR traditional endodontic cavity OR tooth root* OR nonvital* OR endodontic treatment
**#2**	Fracture strength OR Fracture resistance OR tooth fractures OR tooth fractures*
**#3**	Core composite OR fiber post OR restoration OR coronal restoration OR composite core material OR FRC post OR anatomical post OR customized post OR composite resins OR dental restoration OR fiberglass OR post and core technique OR post and core technique* OR fiberglass OR Composite restoration OR dental composite OR dental composite restoration OR Composite resins OR composite resin OR Composite Resins* OR resin OR Composite OR resin based composite OR composite dental resin
**#4**	#1 and #2 and #3

**Table 2:** Demographic and study design data of the included studies.

Study	Composite Resins Used	Core-Build up Composites	Outcomes	Main Result
**Özyürek, 2020** [20]	Filtek Bulk Fill Posterior (3M ESPE, St. Paul, USA)	Clearfil DC Core Plus (Kuraray Medical Inc., Tokyo, Japan)	Fracture strength	The highest resistance to fracture was observed in the samples restored using the RelyX Fiber Post and Filtek Bulk Fill Posterior. Except for the samples restored using FiberSite posts, the fracture strength decreased after the crown replacement. (*p* < 0.05)
**Fráter, 2020** [22]	everX Posterior (GC Europe, Leuven, Belgium) everX Flow (GC Europe, Leuven, Belgium)	Gradia Core (GC Europe, Leuven, Belgium)	Mechanical testing Gap visualization test Microhardness test	The restoration of immature interior teeth with the use of flowable SFRC, as post-core material displayed a promising performance in terms of fatigue resistance and survival.
**Ahmad, 2013** [23]	Composite resin Z100 (3M ESPE, USA)	Alpha-dent (Dental Technologies, USA)	Fracture resistance	There was no significant difference (*p* = 0.233) in fracture resistance between the teeth reinforced with light-polymerizing and auto-polymerizing composite resin. The use of less technique-sensitive auto-polymerizing composite resin had an equivalent beneficial effect on reinforcing weakened roots to the more common light-polymerized composite resin.
**Fráter, 2021** [21]	everX Posterior (GC Europe, Leuven, Belgium) everX Flow (GC Europe, Leuven, Belgium)	Gradia Core Self-Etching Bond (GC Europe, Leuven, Belgium)	Fracture resistance	Regarding fracture pattern, nearly all specimens fractured in a restorable manner Although different FRC post/core systems are available for the restoration of damaged root canal treated anterior teeth, multiple unidirectional FRC posts tend to be a good option when the ferrule is missing
**Panitiwat, 2016** [19]	Tetric N-Ceram (Ivoclar Vivadent, Schaan, Liechtenstein)	Clearfil Photo Core (Kuraray medical, Okayama, Japan) MultiCore Flow (Ivoclar Vivadent, Schaan, Liechtenstein) LuxaCore Z-Dual Automix (DMG, Hamburg, Germany)	Fracture resistance	The fracture resistance was higher in the groups with Clearfil Photo Core and MultiCore Flow, which presented a ranking of the highest values of the materials, showing the same tendency as fracture loads. Among the cores used in this study, the composite core with high filler content tended to enhance the fracture thresholds of teeth restored with fiber posts more than in others.

**Table 3:** Qualitative synthesis (risk of bias assessment).

Study	Specimen Randomization	Single Operator	Operator Blinded	Control Group	Standardized Specimens	Failure Mode	Manufacturer’s Instructions	Sample Size Calculation	Risk of Bias
Özyürek, 2020 [20]	YES	NO	NO	YES	YES	YES	YES	YES	Medium
Fráter, 2020 [22]	YES	NO	NO	YES	YES	YES	YES	NO	Medium
Ahmad, 2013 [23]	YES	NO	NO	YES	YES	NO	YES	NO	Medium
Fráter, 2021 [21]	YES	NO	NO	YES	YES	YES	YES	NO	Medium
Panitiwat,2016 [19]	YES	NO	NO	YES	YES	YES	YES	NO	Medium

## Data Availability

The data that support the findings of this study are available from the corresponding author upon reasonable request.

## Supplementary Materials

The following are available online at https://www.mdpi.com/article/10.3390/polym13142251/s1, Table S1: Search strategy used in SCOPUS; Table S2: Search strategy used in Scielo; Table S3: Search strategy used in Embase; Table S4: Search strategy used in ISI Web of Science.
