# Supplementary material for: Effect of Composite Core Materials on Fracture Resistance of Endodontically Treated Teeth: A Systematic Review and Meta-Analysis of In Vitro Studies
Source: Polymers (Basel). 2021 Jul 9;13(14):2251. doi: 10.3390/polym13142251 (PMC8309392; doi:10.3390/polym13142251)
Supplement: Supplementary file 1 [file polymers-13-02251-s001.zip › polymers-1293580-supplementary.pdf]

polymers

MDPI

Supplementary Materials

Effect of Composite Core Materials on Fracture Resistance of Endodontically Treated Teeth: A Systematic Review and Meta-Analysis of In Vitro Studies

Maciej Zarow [1,*], Marzena Dominiak [2], Katarzyna Szczeklik [3], Louis Hardan [4], Rim Bourgi [4], Carlos Enrique Cuevas-Suárez [5], Juan Eliezer Zamarripa-Calderón [5], Naji Kharouf [6,*] and Dimitar Filtchev [7]

1 Private Practice and Postgraduate Course Center NZOZ SPS Dentist, pl. Inwalidow 7/5, 30-033 Kraków, Poland
2 Department of Dental Surgery, Silesian Piast Medical University, ul. Krakowska 26, 50-425 Wrocław, Poland; Marzena.dominiak@umed.wroc.pl
3 Department of Integrated Dentistry, Jagiellonian University Medical College-Montelupich 4, 31-155 Cracow, Poland; k.szczeklik@uj.edu.pl
4 Department of Restorative Dentistry, School of Dentistry, Saint-Joseph University, Beirut 1107 2180, Lebanon; louis.hardan@usj.edu.lb (L.H.); rim.bourgi@net.usj.edu.lb (R.B.)
5 Dental Materials Laboratory, Academic Area of Dentistry, Autonomous University of Hidalgo State, Circuito Ex Hacienda La Concepción S/N, San Agustín Tlaxiaca 42160, Hgo., Mexico; cecuevas@uaeh.edu.mx (C.E.C.-S.); eliezerz@uaeh.edu.mx (J.E.Z.-C.)
6 Department of Biomaterials and Bioengineering, INSERM UMR_S 1121, Strasbourg University, 67000 Strasbourg, France
7 Department of Prosthetic Dental Medicine, Faculty of Dental Medicine, Medical University of Sofia, 1000 Sofia, Bulgaria; d.filchev@fdm.mu-sofia.bg
* Correspondence: dentist@dentist.com.pl (M.Z.); dentistenajikharouf@gmail.com (N.K.); Tel.: +33-66752-2841 (N.K.)

Citation: Zarow, M.; Dominiak, M.; Szczeklik, K.; Hardan, L.; Bourgi, R.; Cuevas-Suárez, C.E.; Zamarripa-Calderón, J.E.; Kharouf, N.; Filtchev, D. Effect of Composite Core Materials on Fracture Resistance of Endodontically Treated Teeth: A Systematic Review and Meta-Analysis of In Vitro Studies. *Polymers* **2021**, *13*, 2251. https://doi.org/10.3390/polym13142251

Academic Editor: Eija Säilynoja; Sufyan Garoushi and Lippo Lassila

Received: 25 June 2021
Accepted: 8 July 2021
Published: 9 July 2021

Publisher's Note: MDPI stays neutral with regard to jurisdictional claims in published maps and institutional affiliations.

Copyright: © 2021 by the authors. Licensee MDPI, Basel, Switzerland. This article is an open access article distributed under the terms and conditions of the Creative Commons Attribution (CC BY) license (http://creativecommons.org/licenses/by/4.0/).

Table S1. Search strategy used in SCOPUS.

	Search strategy
# 1	TITLE-ABS-KEY("Pulpless tooth" OR "pulpless teeth" OR "root filled tooth" OR "root filled teeth" OR "endodontically treated tooth" OR "endodontically treated teeth" OR "devital tooth" OR "devital teeth" OR "tooth nonvital" OR "root canal treatment" OR "root filling" OR "endodontical treated teeth" OR "endodontics" OR "root canal therapy" OR "tooth root" OR "nonvital" OR "traditional endodontic cavity" OR "endodontic treatment") AND TITLE-ABS-KEY("Fracture strength" OR "Fracture resistance" OR "tooth fractures") AND TITLE-ABS-KEY("Core composite" OR "restoration" OR "coronal restoration" OR "composite core material" OR "composite resins" OR "dental restoration" OR "post and core technique" OR "Composite restoration" OR "dental composite" OR "dental composite restoration" OR "Composite resins" OR "composite resin" OR "resin" OR "Composite" OR "resin based composite" OR "composite dental resin")

Table S2. Search strategy used in Scielo.

	**Search strategy**
# **1**	TS=(Pulpless tooth OR pulpless teeth OR root filled tooth OR root filled teeth OR endodontically treated tooth OR endodontically treated teeth OR devital tooth OR devital teeth OR tooth nonvital OR root canal treatment OR root filling OR endodontical treated teeth OR endodontics OR root canal therapy OR tooth root OR nonvital OR traditional endodontic cavity OR endodontic treatment)
# **2**	TS=(Fracture strength OR Fracture resistance OR tooth fractures)
# **3**	TS=(Core composite OR restoration OR coronal restoration OR composite core material OR composite resins OR dental restoration OR post and core technique OR Composite restoration OR dental composite OR dental composite restoration OR Composite resins OR composite resin OR resin OR Composite OR resin based composite OR composite dental resin)
**#4**	#1 and #2 and #3

Table 3. Search strategy used in Embase.

	**Search strategy**
# **1**	'Pulpless tooth' OR 'pulpless teeth' OR 'root filled tooth' OR 'root filled teeth' OR 'endodontically treated tooth' OR 'endodontically treated teeth' OR 'devital tooth' OR 'devital teeth' OR 'tooth nonvital' OR 'root canal treatment' OR 'root filling' OR 'endodontical treated teeth' OR 'endodontics' OR 'root canal therapy' OR 'tooth root' OR 'nonvital' OR 'traditional endodontic cavity' OR 'endodontic treatment'
# **2**	'Fracture strength' OR 'Fracture resistance' OR 'tooth fractures'
# **3**	'Core composite' OR 'restoration' OR 'coronal restoration' OR 'composite core material' OR 'composite resins' OR 'dental restoration' OR 'post and core technique' OR 'Composite restoration' OR 'dental composite' OR 'dental composite restoration' OR 'Composite resins' OR 'composite resin' OR 'resin' OR 'Composite' OR 'resin based composite' OR 'composite dental resin'
**#4**	#1 and #2 and #3

Table 4. Search strategy used in ISI Web of Science.

	**Scheme .**
# **1**	ALL=(Pulpless tooth OR pulpless teeth OR root filled tooth OR root filled teeth OR endodontically treated tooth OR endodontically treated teeth OR devital tooth OR devital teeth OR tooth nonvital OR root canal treatment OR root filling OR endodontical treated teeth OR endodontics OR root canal therapy OR tooth root OR nonvital OR traditional endodontic cavity OR endodontic treatment)
# **2**	ALL=(Fracture strength OR Fracture resistance OR tooth fractures)
# **3**	ALL=(Core composite OR restoration OR coronal restoration OR composite core material OR composite resins OR dental restoration OR post and core technique OR Composite restoration OR dental composite OR dental composite restoration OR Composite resins OR composite resin OR resin OR Composite OR resin based composite OR composite dental resin)
**#4**	#1 and #2 and #3
